# Assessment of Perceptions of Professionalism Among Faculty, Trainees, Staff, and Students in a Large University-Based Health System

**DOI:** 10.1001/jamanetworkopen.2020.21452

**Published:** 2020-11-23

**Authors:** Dominique A. Alexis, Matthew D. Kearney, J. Corey Williams, Chang Xu, Eve J. Higginbotham, Jaya Aysola

**Affiliations:** 1 Office of Inclusion and Diversity, Perelman School of Medicine, University of Pennsylvania, Philadelphia; 2 Department of Community Health and Prevention, Dornsife School of Public Health, Drexel University, Philadelphia, Pennsylvania; 3 Department of Child and Adolescent Psychiatry and Behavioral Science, Children’s Hospital of Philadelphia, Philadelphia, Pennsylvania; 4 Division of General Internal Medicine, Perelman School of Medicine, University of Pennsylvania, Philadelphia; 5 Leonard David Institute of Health Economics, University of Pennsylvania, Philadelphia; 6 Penn Medicine Center for Health Equity Advancement, Office of the Chief Medical Officer, University of Pennsylvania Health System, Philadelphia

## Abstract

**Importance:**

With a renewed focus on medical professionalism, an opportunity exists to better define its standards and application to meet the needs of an increasingly diverse workforce given the important association between interprofessional behavior and patient care.

**Objective:**

To examine the context of how professionalism is operationalized and perceived in diverse health care work and learning environments.

**Design, Setting, and Participants:**

A qualitative mixed-methods analysis of survey data collected from February to April 2015, was conducted followed by analysis of narrative data collected in June 2017. The setting was 2 health systems and 4 health professional and graduate schools. Participants were faculty, trainees, staff, and students (3506 survey respondents and 52 narratives) affiliated with the University of Pennsylvania and the University of Pennsylvania Health System. Data analysis was conducted in 2018 and 2019.

**Exposures:**

Independent variables included the following respondent characteristics: gender identity, sexual orientation, race/ethnicity, position, generational age group, length of employment at institution, disability status, belief system or religion, and primary site of work or study.

**Main Outcomes and Measures:**

Survey questions were used to assess participants’ perception and experiences of professionalism in the workplace as measured by a 5-point Likert-type scale.

**Results:**

For the survey, there were 3506 respondents from a pool of 18 550 potential respondents (18.9% response rate). Of 3506 survey respondents, 2082 of 3231 (64.4%) were women, 331 of 3164 (10.5%) identified as gender or sexual minority groups, and 360 of 3178 (11.3%) were non-Hispanic Black individuals. In adjusted analyses, women compared with men (adjusted odds ratio [aOR], 1.8; 95% CI, 1.4-2.3) and Asian individuals (aOR, 2.0; 95% CI, 1.7-2.3) and Hispanic individuals (aOR, 2.0; 95% CI, 1.4-2.7) compared with non-Hispanic White individuals were more likely to value institutional professionalism. In addition, gender identity and sexual minority groups compared with heterosexual respondents (aOR, 1.5; 95% CI, 1.2-1.8) and non-Hispanic Black individuals compared with non-Hispanic White individuals (aOR, 1.3; 95% CI, 1.2-1.4) were statistically significantly more likely to consider changing jobs because of unprofessional behavior at work. The qualitative analysis of narratives revealed that marginalized populations (including but not limited to women, gender and sexual minority groups, racial/ethnic minority groups, those who identify as having a disability, and religious minority groups) reported (1) greater infringements on their professional boundaries, as well as increased scrutiny over their professional actions, and (2) a tension between inclusion vs assimilation.

**Conclusions and Relevance:**

The findings of this study highlight the need for health care organizations to revisit how they define and operationalize professionalism to improve inclusivity.

## Key Points

**Question:**

Are current professionalism standards in the health care workforce and learning environments inclusive to all groups?

**Findings:**

We conducted a mixed-methods analysis using survey data (n = 3506) and qualitative narrative data (n = 52) and found that marginalized groups reported experiencing professionalism differently as evidenced by statistically significant differences in responses, as well as 2 themes that reveal marginalized populations’ perceptions of being subject to greater infringements on their professionalism boundaries, increased scrutiny over their professional actions, and pressure to conform.

**Meaning:**

Understanding how marginalized groups perceive professionalism in health system work and learning environments suggests that the current standards of professionalism need to be evaluated and redefined to improve the culture of academic medicine.

## Teaser

This qualitative study used a mixed-methods approach to analyze survey and narrative data on how professionalism standards are operationalized and perceived in diverse health care work and learning environments.

## Introduction

Professionalism is an important unifying principle in medicine and has been historically described as “the basis of medicine’s contract with society.”^1^^(p243)^ This contract for professionalism gained prominence in the 1990s,^2,3,4,5^ when members of the medical field agreed on and promised to uphold a set of “ethical values and competency standards” that “the public and individual patients can and should expect from medical professionals.” Thereafter, medical professionalism was implemented as a core competency for undergraduate medical education and graduate medical education to govern how members of the profession conduct themselves in public, be it with patients or each other.^6,7,8^ Despite these efforts, the medical field lacks a concise, unifying, and operational definition of professionalism. Professionalism remains a fluid and contextual notion that is thereby often misused or overused,^9,10,11,12^ with greater emphasis in practice placed on the delivery of patient care rather than interprofessionalism within the workplace.^13^

Professionalism is a historical construct. The term *professionalism* is often used to describe a broad set of behavior, dress, language, affect, and hierarchies that are deemed appropriate in medicine. Historically, medical institutions did not have women and members of minority groups in positions of leadership and authority, which resulted in professional norms largely being centered around White male identity and the broader cultural norms associated with whiteness.^14,15,16^ The current conceptualization of what is deemed professional often does not include diverse groups and thereby can be noninclusive or discriminatory.^14^ For example, these cultural norms may result in perceiving the way that certain groups wear their hair or speak as unprofessional.^15^ Thus, there is a need to evaluate how professionalism standards are applied in different populations and cultures, as well as to reexamine how professionalism is defined given the increasing diversity of the health care workforce.^16^ Consensus is lacking on how to effectively integrate inclusion into developing and operationalizing professionalism within health care work and learning environments.^13^ Therefore, the objective of this study was to examine the perceptions and experiences of professionalism among faculty, trainees, staff, and students in diverse health care work and learning settings.

## Methods

We conducted a qualitative study using a mixed-methods approach to characterize perceptions of professionalism with data from 2 distinct sources. First, a quantitative analysis of existing survey data^17^ was performed, followed by a qualitative analysis of a subset of narrative data collected in June 2017. The University of Pennsylvania Institutional Review Board approved our study protocol and the recruitment language that detailed public use of only deidentified narratives. Participants consented for public use of their anonymized data by submitting a response, as indicated in the instructions given in the email. We followed the Standards for Reporting Qualitative Research (SRQR) reporting guideline in this study.

### Quantitative Analysis

We analyzed a subset of data pertaining to professionalism from the Diversity Engagement Survey^17^ collected from February to April 2015 via email by DataStar^18^ to faculty, trainees, staff, and students at 2 health systems (the Children’s Hospital of Pennsylvania and the Corporal Michael J. Crescenz VA Medical Center) and 4 health professional and graduate schools (Perelman School of Medicine, University of Pennsylvania School of Nursing, University of Pennsylvania School of Dental Medicine, and University of Pennsylvania School of Social Policy & Practice) that are all affiliated with the University of Pennsylvania and the University of Pennsylvania Health System. Survey completion was voluntary and without incentives. Three email reminders were sent to maximize response rates. Further survey methods are described elsewhere.^19^

### Independent Variables

The following self-reported respondent characteristics were evaluated: gender identity, sexual orientation, race/ethnicity, position, generational age group, length of employment at institution, disability status, belief system or religion, and primary site of work or study (where one spends most time working).

### Dependent Variables

The key outcomes of interest were respondents’ perceptions of professionalism in the workplace captured by responses to the following 3 statements: (1) “I have considered changing jobs due to inappropriate, disruptive, or unprofessional behavior by a coworker or supervisor.” (2) “I value institutional initiatives, policies, and/or educational resources related to professional behavior in the workplace.” (3) “My institution supports a culture of professionalism.” The 5-point Likert-type scale response options to the statements were 5 (strongly agree), 4 (agree), 3 (neither agree nor disagree), 2 (disagree), or 1 (strongly disagree).

### Statistical Analysis

We examined the association between the self-identified respondent characteristics (independent variables) and each of the 3 professionalism statements. After examining the distributions of the dependent variables and performing sensitivity analyses evaluating the outcome as continuous vs binary, the outcomes were dichotomized for ease of interpretation. Responses were categorized as either affirmative (5 [strongly agree] or 4 [agree]) or not affirmative or neutral (3 [neither agree nor disagree], 2 [disagree], or 1 [strongly disagree]). We fitted multivariable logistic regression models to estimate the associations of respondent characteristics with our binary professionalism outcomes, adjusting for the independent variables specified previously, using generalized estimating equations with robust SEs to account for clustering by site. Two-tailed *P* values and 95% CIs are reported for all covariates. *P* < .05 was considered statistically significant.

### Qualitative Analysis of Narratives

#### Study Design

To better understand the factors that may be contributing to differences in responses to professionalism questions, we analyzed anonymous narratives collected in June 2017 through a campaign entitled “Please Tell Us Your Story of Inclusion.” Across 2 health systems and 4 health professional and graduate schools, faculty, trainees, staff, and students submitted solicited personal narratives (n = 315) in response to 2 stimulus questions designed to assess experiences with professionalism and inclusion at workplace or school. Content analysis was conducted on completed narratives stripped of any identifiers, producing a thematically coded data set.^20,21^ Further methods are reported elsewhere.^22^

#### Sampling and Analysis

There was no overt code for professionalism in the parent study^22^; therefore, we began by identifying narratives that mentioned the term *professional* or *professionalism* (n = 13). Then, narratives that mentioned these terms were examined to identify salient clusters of co-occurring themes, yielding the following 3 previously coded themes: hierarchy, interpersonal dynamics, and social norms. Finally, the remaining 302 narratives from the parent study were reviewed to identify other narratives coded for these 3 themes (n = 39), producing a final sample of 52 narratives associated with professional behavior in the workplace or lack thereof.

Throughout a series of in-person meetings, a codebook was iteratively developed until reaching thematic saturation.^21^ Next, the research team (D.A.A., M.D.K., and J.A.) reviewed the final group of narratives to identify salient patterns associated with professionalism, its causes, and its consequences. We examined these patterns by respondent characteristics to assess differences by subpopulations. We specifically focused on comparisons between those subgroups that would be classified as marginalized, defined as subgroups systematically excluded from mainstream social, economic, cultural, or political life, with subsequent unequal power in group relationships within society.^23^ Marginalized populations would include but are not necessarily limited to women, gender and sexual minority groups, racial/ethnic minority groups, those that identify as having a disability, and religious minority groups.

## Results

### Quantitative Analysis

From a pool of 18 550 potential respondents, there were 3506 survey respondents (18.9% response rate). Of those 3506 respondents, 2082 of 3231 (64.4%) were female, 331 of 3164 (10.5%) identified as LGBTQ (lesbian, gay, bisexual, transgender, and/or queer), and 360 of 3178 (11.3%) were non-Hispanic Black individuals (Table 1 and Table 2).

**Table 1.** Baseline Characteristics of Survey Respondents

Variable	No. (%) (N = 3506)
Gender identity	
Male	1134 (35.1)
Female	2082 (64.4)
Transgender/queer^a^	15 (0.5)
Sexual orientation	
Heterosexual	2833 (89.5)
LGBTQ^b^	331(10.5)
Race/ethnicity	
Non-Hispanic White	2077 (65.4)
Non-Hispanic Black	360 (11.3)
Asian	358 (11.3)
Hispanic/Latino	206 (6.5)
Multiple races/ethnicities	89 (2.8)
Other races/ethnicities^c^	88 (2.8)
Position	
Executive	122 (3.8)
Faculty or physician	759 (23.5)
House staff or trainee^d^	238 (7.4)
Staff^e^	
Graduate or postdoctoral student	648 (20.0)
Undergraduate student	64 (2.0)
Other position	14 (0.4)
Length of employment at institution, y	
<1	521 (16.0)
At least 1 but <5	1217 (37.5)
At least 5 but <10	508 (15.6)
≥10	1002 (30.9)
Disability status	
Yes	64 (2.0)
No	3002 (93.3)
Declined to answer	153 (4.8)
Belief system or religion	
Judeo-Christian	1449 (45.4)
Non–Judeo-Christian^f^	1476 (46.2)
Declined to answer	267 (8.4)
Primary site of work or study	
Children’s Hospital of Philadelphia	465 (14.3)
Penn Dental Medicine	136 (4.2)
University of Pennsylvania affiliated hospitals^g^	907 (28.0)
School of Nursing	117 (3.6)
School of Social Policy & Practice	90 (2.8)
Perelman School of Medicine	1236 (38.1)
Other	292 (9.0)
Primary language	
English	2964 (92.1)
Non-English	256 (7.9)

Abbreviation: LGBTQ, lesbian, gay, bisexual, transgender, and/or queer.

^a^ Includes transgender, other gender identity, and do not identify as male or female.

^b^ Includes LGBTQ and other sexual orientations.

^c^ Includes Native American/Alaskan native, Pacific Islander, and other races/ethnicities.

^d^ Includes interns, residents, fellows, and postdoctoral fellows.

^e^ Includes medical, dental, graduate, doctoral, and postdoctoral students.

^f^ Includes all other categories (and Jewish).

^g^ Includes Ruth & Raymond Perelman Center for Advanced Medicine, Hospital of the University of Pennsylvania, Penn Presbyterian Medical Center, Pennsylvania Hospital, and Philadelphia VA Medical Center.

**Table 2.** Baseline Characteristics of Narrative Respondents

Variable	No. (%) (N = 52)
Gender identity	
Male	15 (28.8)
Female	33 (63.5)
Other gender identity	2 (3.8)
Do not identify as male or female	1 (1.9)
Declined to answer	1 (1.9)
Sexual orientation	
Heterosexual	40 (76.9)
Lesbian or gay or homosexual	5 (9.6)
Bisexual	2 (3.8)
Other sexual orientation	2 (3.8)
Declined to answer	3 (5.8)
Race/ethnicity	
Non-Hispanic White	28 (53.8)
Non-Hispanic Black	7 (13.5)
Asian	5 (9.6)
Hispanic/Latino	4 (7.7)
Other races/ethnicities^a^	7 (13.5)
Declined to answer	1 (1.9)
Position	
Staff	18 (34.6)
Faculty or physician	14 (26.9)
Staff-manager level	7 (13.5)
House staff or trainee	7 (13.5)
Graduate or postdoctoral student	5 (9.6)
Undergraduate student	1 (1.9)
Length of employment at institution, y	
<1	5 (9.6)
At least 1 but <2	3 (5.8)
At least 2 but <5	14 (26.9)
At least 5 but <10	11 (21.2)
≥10	19 (36.5)
Disability status	
No	41 (78.8)
Declined to answer	6 (11.5)
Yes	4 (7.7)
Belief system or religion	
Judeo-Christian	26 (50.0)
None	12 (23.1)
Hindu	4 (7.7)
Jewish	3 (5.8)
Other	3 (5.8)
Declined to answer	2 (3.8)
Unitarian Universalist	2 (3.8)
Primary site of work or study	
Perelman School of Medicine	25 (48.1)
Hospital of the University of Pennsylvania	9 (17.3)
Other	6 (11.5)
Ruth & Raymond Perelman Center for Advanced Medicine	5 (9.6)
School of Nursing	3 (5.8)
Penn Dental Medicine	1 (1.9)
Penn Presbyterian Medical Center	1 (1.9)
Pennsylvania Hospital	1 (1.9)
Philadelphia VA Medical Center	1 (1.9)
Primary language	
English	45 (86.5)
Spanish	3 (5.8)
Declined to answer	1 (1.9)
Hindustani	1 (1.9)
Other	1 (1.9)
Russian	1 (1.9)

^a^ Includes Native American/Alaskan native, Pacific Islander, and other races/ethnicities.

There were statistically significant unadjusted differences in responses to all 3 professionalism statements by respondent gender identity, sexual orientation, race/ethnicity, and position. Table 3 lists all unadjusted comparisons by respondent characteristics. In response to the statement “I have considered changing jobs due to inappropriate, disruptive, or unprofessional behavior by a co-worker or supervisor,” 14.3% of respondents that identified as female, 14.8% of non-Hispanic Black individuals, 15.5% of LGBTQ individuals, and 16.3% of staff agreed in contrast to 7.9% of males (*P* < .001), 11.2% of non-Hispanic Whites (*P* = .002), 11.4% of heterosexuals (*P* = .007), and 10.4% of faculty (*P* < .001), respectively. In response to the statement “I value institutional initiatives, policies, and/or educational resources related to professional behavior,” 52.3% of females, 54.1% of non-Hispanic Blacks, and 54.3% of staff agreed more compared with 45.4% of males (*P* < .001), 49.0% of non-Hispanic Whites (*P* = .002), and 47.3% of faculty (*P* < .001), respectively. Last, in response to “My institution supports a culture of professionalism,” 54.2% of females, 58.2% of non-Hispanic Blacks, 54.8% of LGBTQ, and 55.6% of staff agreed in contrast to 50.0% of males (*P* < .001), 54.2% of non-Hispanic Whites (*P* < .001), 52.6% of heterosexuals (*P* < .001), and 49.2% of faculty (*P* < .001), respectively. Table 3 lists the number and percentage of the remaining respondents for each professionalism question from our unadjusted analyses.

**Table 3.** Unadjusted Rates of Agreement With Professionalism Statements by 3506 Survey Respondent Characteristics^a^

Variable	“I have considered changing jobs due to inappropriate, disruptive, or unprofessional behavior by a coworker or supervisor”		“I value institutional initiatives, policies, and/or educational resources related to professional behavior in the workplace”		“My institution supports a culture of professionalism”	
	Agree, No. (%)	*P* value	Agree, No. (%)	*P* value	Agree, No. (%)	*P* value
Sex identity						
Male	95 (7.9)	<.001	544 (45.4)	<.001	599 (50.0)	<.001
Female	323 (14.3)		1181 (52.3)		1226 (54.2)	
Sexual orientation						
Heterosexual	345 (11.4)	.007	1513 (50.0)	.30	1593 (52.6)	<.001
LGBTQ^b^	55 (15.5)		180 (50.6)		195 (54.8)	
Race/ethnicity						
Non-Hispanic White	253 (11.2)	.002	1106 (49.0)	.07	1183 (54.2)	<,001
Asian	54 (14.6)		213 (57.4)		211 (56.9)	
Non-Hispanic Black	54 (14.8)		198 (54.1)		213 (58.2)	
Other races/ethnicities^c^	17 (18.5)		42 (45.7)		46 (50.0)	
Hispanic	21 (9.6)		96 (44.2)		97 (44.7)	
Multiple races/ethnicities	6 (6.1)		43 (43.4)		49 (49.5)	
Position						
Executive	9 (6.9)	<.001	49 (37.4)	<.001	62 (44.3)	<.001
Faculty or physician	88 (10.4)		402 (47.3)		418 (49.2)	
Fellow^d^	40 (9.9)		178 (44.1)		199 (49.3)	
Other position^e^	3 (20.0)		9 (60.0)		9 (60.0)	
Staff^f^	233 (16.3)		779 (54.3)		799 (55.6)	
Student^g^	43 (6.9)		307 (49.0)		335 (53.4)	

Abbreviation: LGBTQ, lesbian, gay, bisexual, transgender, and/or queer.

^a^ Seven respondents did not answer any of the questions about agreement with the statements.

^b^ Includes LGBTQ and other sexual orientations.

^c^ Includes Native American/Alaskan native, Pacific Islander, and other races/ethnicities (reflects both unspecified and free-text specified responses). The most common specified responses were Middle Eastern/Arab and South Asian/Asian Indian.

^d^ Includes resident/fellow/intern/postdoctoral and PhD students.

^e^ Includes outpatient practices, satellite sites, and/or other services.

^f^ Includes staff and staff-manager level.

^g^ Includes medical, dental, graduate, doctoral, and postdoctoral students.

In adjusted models, respondents who self-identified as female, LGBTQ, non-Hispanic Black individuals, and staff compared with male, heterosexual, non-Hispanic White individuals, and faculty were statistically significantly more likely to report considering changing jobs because of unprofessional behavior. For example, gender and sexual minority groups compared with heterosexual respondents (aOR, 1.5; 95% CI, 1.2-1.8) and non-Hispanic Black individuals compared with non-Hispanic White individuals (aOR, 1.3; 95% CI, 1.2-1.4) were statistically significantly more likely to consider changing jobs because of unprofessional behavior at work. Women compared with men (adjusted odds ratio [aOR], 1.8; 95% CI, 1.4-2.3) and Asian individuals (aOR, 2.0; 95% CI, 1.7-2.3) and Hispanic individuals (aOR, 2.0; 95% CI, 1.4-2.7) compared with non-Hispanic White individuals were more likely to value institutional professionalism. No statistically significant adjusted differences were found among respondents who agreed with the statement “My institution supports a culture of professionalism.” Table 4 lists the odds ratios and 95% CIs for all of the adjusted comparisons.

**Table 4.** Adjusted Rates of Agreement With Professionalism Statements by Respondent Characteristics

Variable	“I have considered changing jobs due to inappropriate, disruptive, or unprofessional behavior by a coworker or supervisor”		“I value institutional initiatives, policies, and/or educational resources related to professional behavior in the workplace”		“My institution supports a culture of professionalism”	
	Agree, aOR (95% CI)	*P* Value	Agree, aOR (95% CI)	*P* Value	Agree, aOR (95% CI)	*P* Value
Gender identity						
Transgender/queer vs male	1.5 (0.4-6.0)	.50	0.4 (0.2-1.1)	.09	0.6 (0.2-1.4)	.20
Female vs male	1.3 (1.1-1.5)	.02	1.8 (1.4-2.3)	<.001	0.7 (0.5-0.9)	.03
Sexual orientation						
LGBTQ vs heterosexual^a^	1.5 (1.2-1.8)	<.001	0.8 (0.7-0.9)	.004	0.5 (0.4-0.7)	<.001
Race/ethnicity						
Non-Hispanic other races/ethnicities vs non-Hispanic White	1.8 (1.2-2.5)	.002	1.1 (0.6-2.1)	.80	0.7 (0.5-0.9)	.03
Non-Hispanic multiple races/ethnicities vs non-Hispanic White	0.9 (0.6-1.2)	.50	1.4 (1.0-1.8)	.05	0.8 (0.5-1.2)	.20
Non-Hispanic Black vs non-Hispanic White	1.3 (1.2-1.4)	<.001	1.2 (0.9-1.7)	.30	0.9 (0.8-1.1)	.30
Non-Hispanic Asian vs non-Hispanic White	1.4 (0.9-2.0)	.10	2.0 (1.7-2.3)	<.001	1.2 (1.2-1.4)	.002
Hispanic vs non-Hispanic White	1.0 (0.6-1.6)	.90	2.0 (1.4-2.7)	<.001	1.5 (0.9-2.5)	.10
Position						
Student vs faculty/physician^b^	0.7 (0.4-1.1)	.10	1.0 (0.6-1.7)	.90	1.1 (0.8-1.4)	.60
Staff vs faculty/physician^c^	1.6 (1.2-7.9)	<.001	1.6 (1.2-2.1)	.002	1.0 (0.8-1.3)	.90
Other vs faculty/physician	2.0 (0.6-6.0)	.20	2.0 (0.2-21.0)	.50	1.2 (0.3-4.9)	.80
Fellow vs faculty/physician^d^	1.0 (0.7-1.6)	.90	0.9 (0.6-1.2)	.50	0.9 (0.6-1.2)	.40
Executive vs faculty/physician	0.9 (0.5-1.7)	.80	1.3 (0.7-2.8)	.40	2.0 (0.8-5.1)	.10
Generational age group						
1965-1980 vs 1981-2000	1.3 (1.0-1.6)	.02	0.8 (0.7-1.1)	.30	0.6 (0.5-0.8)	<.001
1945-1964 vs 1981-2000	1.2 (0.9-1.5)	.30	1.3 (0.8-2.2)	.30	0.6 (0.4-1.1)	.10
1922-1944 vs 1981-2000	0.3 (0.1-0.5)	<.001	1.3 (0.6-3.0)	.50	4.0 (1.2-13.6)	.03

Abbreviations: aOR, adjusted odds ratio; LGBTQ, lesbian, gay, bisexual, transgender, and/or queer.

^a^ LGBTQ includes LGBTQ and other sexual orientations.

^b^ Student includes medical, dental, graduate, doctoral, and postdoctoral students.

^c^ Staff includes staff and staff-manager level.

^d^ Fellow includes resident/fellow/intern/postdoctoral and PhD students.

### Qualitative Analysis

Among the 52 narratives, 33 respondents (63.5%) self-identified as female, 7 (13.5%) as LGBTQ, 28 (53.9%) as non-Hispanic White, and 7 (13.5%) as non-Hispanic Black (Table 2). Two themes emerged from the narratives. Marginalized populations reported (1) greater infringements on their professional boundaries, as well as increased scrutiny over their professional actions, and (2) a tension between inclusion vs assimilation. In addition, analyses revealed that religious minority groups, specifically those not identifying as Judeo-Christian, struggled in similar ways. We found the same patterns as described above.

### Theme 1: Marginalized Populations Experience Professionalism Differently

#### Infringements on Professional Boundaries

Many narrators who self-identified as members of marginalized populations expressed greater infringement on their professional boundaries during interactions at work or learning environments. The infringements reported ranged from microaggressions to blatant racism, sexism, xenophobia, homophobia, and harassment. Whether subtle or aggressive, the infringements on professional boundaries disproportionately impacted women and gender, sexual, and racial/ethnic minority groups (Table 5). One respondent stated: “The physician grabbed my right forearm and pulled it towards the patient. The physician asked the patient ‘does it [the patient’s color of diarrhea] look like her arm or is it lighter?’…The physician chuckled and said ‘your skin is the perfect color for this job’….”

**Table 5.** Key Professionalism Themes and Representative Quotations

Variable	Representative quotations
**Theme 1: marginalized populations experience professionalism differently**	
Greater infringements on their professional boundaries	“I met with [a] senior male faculty member. …I was rather pregnant at the time. He spent a fair amount of the meeting talking to me about my plans to breastfeed and how that would impact me and my life (eg, I wouldn’t have any freedom because I would always ‘have a baby attached to my breast’). Needless to say, it made me very uncomfortable.”
Increased scrutiny over their professional actions	“There are some examples, but they are subtle yet apparent. One thing I have noticed is that…residents of color seem to get criticized for things that the majority do not, even if they do the same things. There is this microscope that is applied to them which…is subtle, yet present.” “I have witnessed a few instances where women or non-White students or employees were treated in a disrespectful or discriminatory [manner], and I conclude that, despite the rules and policies, it all depends on the particular individual you end up dealing with.”
**Theme 2: tension exists between inclusion vs assimilation**	
Pressure to conform	“I am a heavily tattooed person. [T]he comments, stares, and otherwise general dismissal of me as a colleague based simply on my tattoos has become a daily struggle.” “Many internationals compromise personal dignity and values so they don’t get kicked out, but ultimately it only promotes intolerance and behavior aimed at silencing their voices and opinions rather than making them feel included and welcome.” “Was asked to cover for my colleagues during Christmas or Jewish [H]igh [H]olidays and I was happy to do so. In later years…I have asked for time off during my own religious holidays. This has been met with polite ignorance and an unconscious devaluation of non–Judeo-Christian traditions.” “I think about conversations about certain students who ‘don’t fit’ when really we are describing them as different.”

Another respondent noted: “I was told to consider living in ‘not so nice neighborhoods’ and not to ‘eat and drink out’ and to commute by bike…. I was also told not to invite my parents to care for our baby since my wife will be home anyways and then I won’t need a larger apartment so I can fit all my expenses in my current salary. I felt marginalized and I felt that [my superior’s] behavior was excessively intrusive.”

Narratives consistently described that women and minority groups navigating health care work and learning environments were subject to multiple experiences of unprofessionalism because of their identity. One narrator expressed: “I have felt a gender and age bias on several occasions…. When I am working with older men…I am spoken down, disrespected, or outright ignored. I am generally made to feel that my thoughts are invalid or not helpful….”

#### Scrutiny Over Professional Actions

Many narratives stated that professional standards were applied differently to certain groups, and those groups perceived that they were subject to greater scrutiny. Examples of differential treatment varied in degree (Table 5). One respondent disclosed: “…[E]very time the [minority group] resident came late to rounds, even if he/she was accompanying a patient to a test, it was documented in writing. It didn’t matter if White residents came later to rounds or conferences; their lateness was never documented.”

Another participant wrote: “I have experienced and witness leadership in our division systematically do this across ethnic lines, where minorities and outsiders have to work much harder to prove themselves and get treated with respect.”

Yet another narrative stated: “The candidate we knew is a woman of color from a working-class background who has some idiomatic speech patterns and has mostly learned in the job, while the candidates we were sent with a real push to hire instead were White, from middle-class backgrounds with speech patterns of that cultural group and more formal education (but less relevant on-the-ground experience). It gave a strong sense that looking/speaking like our current entirely White administration was valued over the known skills of our candidate.”

### Theme 2: Tension Between Inclusion vs Assimilation

We found that respondents perceived the need to assimilate into their environments as opposed to the workplace adopting an inclusive environment. Many respondents perceived pressure to conform to the dominant norms of their work environment or their positions would be at stake. This pressure often meant adhering to norms associated with White identity. Words frequently used in the narratives were “fit,” “pressure,” and “individuality.” For example, a respondent stated how there was a “strong push toward uniformity at the expense of individuality and the energy that comes from the interaction of people of different backgrounds.”

Narrators expressed that they did not perceive being welcomed for their differences but rather isolated because of them regardless of their hard work and contributions to their department or team. One narrator noted: “I don’t feel welcomed here. Even in the URM [underrepresented minority] groups like LMSA [Latino Medical Student Association] or SNMA [Student National Medical Association], I felt the pressure to be ‘Whiter’ and not allowed to feel comfortable in my own skin/language/culture. I don’t feel included, but instead feel the pressure to become White.”

Another narrator expressed: “Many internationals compromise personal dignity and values so they don’t get kicked out, but ultimately it only promotes intolerance and behavior aimed at silencing their voices and opinions rather than making them feel included and welcome” (Table 5).

Narratives also highlighted the tension between perceiving pressure to conform vs leaving the institution. For example, one narrator expressed: “As a queer person of color…[I am mostly made to] feel ‘other’ in one way or another! That is one of many reasons why I will be leaving soon…it’s too much.”

Such external pressure to conform often manifested in reported disciplinary action. One narrative stated: “I was called into a standard/probation meeting on a Muslim learner…. [I]t was commented on the case that the individual ‘would not even eat our foods’ as a reason to question their academic performance…as ‘the learner only ate halal foods’.”

Narratives consistently described use of the word “fit” to suggest that individuality was not appreciated or sought out. For example, a respondent stated that the institution is “inclusive if you fit the mold of what is expected.” Another noted: “First I watched the residency director tell their faculty colleagues that a resident of color was just a bad fit in their residency. They didn’t say why, just a bad fit.”

## Discussion

Professionalism in medicine has been championed as a contract between all members of the profession that governs the membership’s public conduct, be it with patients or each other.^1,2,3,4,5^ The objective of this mixed-methods analysis was to examine the perspectives of a diverse set of faculty, clinical providers, trainees, staff, and students to assess if there is a need to revisit this contract. We found that individuals self-identifying as female, underrepresented racial/ethnic minority groups,^24^ gender and sexual minority groups, and staff value professionalism equally or even greater than their counterparts, yet they perceive or experience it differently within their organizations. The greater value women and underrepresented minority groups place on professionalism may stem from what they perceive to be lacking in their work environment and gaps that they perceive between institutional values and their lived experiences. This study used the Diversity Engagement Survey, a validated survey instrument developed by Person et al,^17^ to assess how organizations effectively engage their diverse membership. Our study builds on this prior work,^17,19,21,25^ with an in-depth look into how organizations engage diversity when operationalizing professionalism and reveals the need to revisit how we both define and apply professionalism in medicine.

The narrative themes augmented and reinforced the quantitative findings that women, staff, racial/ethnic, and sexual and gender minority groups were statistically significantly more likely to consider changing jobs due to unprofessional behavior that manifests as bias and as bias and discrimination. These groups reported greater infringements on their professional boundaries and increased scrutiny over their professional actions within the workplace. Applying standards inequitably can result in disadvantages for some, thereby creating advantages for others, resulting in perceptions of isolation and lack of engagement by those individuals. As noted by Nivet,^26^ the many disadvantaged positions that minority group faculty members experience compared with others have been established through years of systematic segregation, discrimination, tradition, culture, and elitism in academic medicine.^27,28,29,30,31^ This observation is also applicable to other historically marginalized populations, including women, LGBTQ persons, and individuals born outside of the United States.^27,32,33,34,35,36^

Our findings highlight the tension between inclusion and assimilation. The need to conform to a set of noninclusive professional standards emerged as a key challenge for women and minority groups (gender, sexual, and racial/ethnic) alike. Seminal work in understanding the nature of belonging or inclusion by Brown^37,38,39^ describes inclusivity as the opposite of conforming or assimilation. By this definition, professionalism standards are not inclusive when underrepresented members of a profession have to assimilate or conform in an effort to adhere to them. The concept of covering has also been used to describe this same notion. Covering, a word coined by Erving Goffman, details how individuals with known stigmatized identities perceive greater pressure and put more effort into downplaying those identities to assimilate better into majority groups.^40,41^ Understanding how and why women and minority groups cover and assimilate can help break down barriers and lead to a culture of inclusion and authenticity.

In addition, findings of the present study suggest that the medical field must revisit and expand professionalism standards to ensure that the increasingly diverse membership helps to develop and agree on a “belief system in which group members declare shared competency standards and ethical values they promise to uphold in their work.”^3^ We believe that such competency standards and ethical values must include fair treatment of patients and colleagues regardless of position or personal characteristics, such as gender identity, sexual orientation, and race/ethnicity. However, this goal is not enough. Health care organizations and medical institutions can also engage their diverse membership to ensure professionalism standards are equitably applied.

Professionalism and the development of associated standards were devised with a narrow lens given the historical homogeneity of academic medicine.^26,42,43,44,45^ As a result, there is some evidence that standards are vulnerable to being mobilized with a much greater degree of strictness among marginalized groups.^22^ The concept of professionalism itself can center norms associated with a singular majority culture and inadvertently marginalize minority groups. The findings of the present study reveal how medical professionalism can be displayed through the unequal application of standards, also referred to as being “weaponized.” This weaponization, as noted by Lee,^46^ can damage individuality and identity and cause conformity, shrink efforts to increase the diversity of the workforce, create harmful environments, and force marginalized populations to assimilate in an attempt to be included.^40,41^ Health care professionals, organizations, and medical institutions can ensure that the standards collectively set to govern conduct with patients and each other are not weaponized to pressure members of our profession to assimilate.

An increasing number of studies report that women and minority groups have higher attrition rates than majority groups.^30,47,48,49,50,51,52,53,54,55,56,57,58,59,60,61^ The findings of the present study build on this work by revealing why marginalized groups may seek employment elsewhere. Departments and divisions have their own unspoken rules, norms, policies, and leadership styles that can create different daily workplace cultures within an institution.^62^ This study supports the need for institutional policy adjustments pertaining to professionalism. There remains a need to create environments where individuals are comfortable to be themselves and express a range of cultural practices and traditions. Such inclusive environments may help mitigate factors, such as avoidance or the persistent need to conform, that if left unchecked can lead to attrition, disillusionment, and less organizational commitment, in addition to the personal trauma of discrimination.^22,63,64,65,66^

### Strengths and Limitations

A notable strength of this study is the application of a mixed-methods approach to further understand the intersection of professionalism and inclusion. We acknowledge some limitations to this study. The findings may not be generalizable to other study settings. However, our data are from a diverse regional set of hospitals and health science schools in New Jersey, Pennsylvania, and Delaware. The quantitative survey assessment of respondents’ belief system or religion was limited to 2 categories (Judeo-Christian or not); however, the qualitative assessment captured a broader array of categories. In addition, the survey administration method allowed for individuals present on more than 1 listserve to be counted more than once in our sampling population and may have resulted in an underestimation of the response rate. Individuals completing the survey more than once would counter this limitation. However, we believe individuals would not be inclined to complete multiple surveys given the survey burden and lack of monetary incentive.^19^ Furthermore, the response rates by characteristic are at par with or exceed those of national benchmark response rates to this survey^67^ (eAppendix in the Supplement). In addition, the study data may have been subject to selection bias. However, the analysis by design benefits from responses of individuals motivated to provide their perceptions.

## Conclusions

This study highlights the need for commitment from key stakeholders to contribute to a paradigm shift in how professionalism standards are developed and applied. From a business standpoint, retention decreases the cost of recruitment, training, and attrition. However, we believe that to retain diverse faculty, trainees, and staff, these groups need to perceive that they are included and valued. The definition and operationalization of medical professionalism standards within academic centers and health care work environments is paramount to building a more inclusive culture.^16,68,69^ An environment that engages diverse individuals and perspectives ultimately impacts health system value through high-quality patient care, novel research, and culturally mindful education for the next generation of health care professionals.^13,70,71^ Examining professionalism through the lens of inclusion provides an opportunity to create academic health care systems where all members, as their authentic selves, perceive that they are welcomed and valued.

## Notes

Supplement. **eAppendix.** Response Rates by Characteristics Benchmarked Against Other Institutions That Administered the Diversity Engagement Survey
