# Supplementary material for: Assessment of Perceptions of Professionalism Among Faculty, Trainees, Staff, and Students in a Large University-Based Health System
Source: JAMA Netw Open. 2020 Nov 23;3(11):e2021452. doi: 10.1001/jamanetworkopen.2020.21452 (PMC7684446; doi:10.1001/jamanetworkopen.2020.21452)
Supplement: Supplement. — eAppendix. Response Rates by Characteristics Benchmarked Against Other Institutions That Administered the Diversity Engagement Survey [file jamanetwopen-e2021452-s001.pdf]

Supplementary Online Content

Alexis DA, Kearney MD, Williams JC, Xu C, Higginbotham EJ, Aysola J. Assessment of perceptions of professionalism among faculty, trainees, staff, and students in a large university-based health system. *JAMA Netw Open*. 2020;3(11):e2021452. doi:10.1001/jamanetworkopen.2020.21452

eAppendix. Response Rates by Characteristics Benchmarked Against Other Institutions That Administered the Diversity Engagement Survey

This supplementary material has been provided by the authors to give readers additional information about their work.

© 2020 Alexis DA et al. *JAMA Network Open*.

eAppendix. Response Rates by Characteristics Benchmarked Against Other Institutions That Administered the Diversity Engagement Survey

	**UPenn (2015)**	**Benchmark**
	**N (%)**	**N (%)**
**Respondents**	**3506 (100)**	**13694 (100)**
**Gender**		
Male	1197 (34)	4479 (33)
Female	2255 (64)	8811 (64)
**Race/Ethnicity**		
White	2409 (69)	9789 (71)
Black/African-American	379 (11)	1134 (8)
Hispanic/Latino(a)	132 (4)	738 (5)
Asian	376 (11)	1002 (7)
Other	117 (3)	487 (4)
**Sexual Orientation**		
Heterosexual	3037 (87)	11846 (87)
LGBTQ[a] or Other	358 (10)	950 (7)
Missing/Refused to Answer	111 (3)	898 (7)

© 2020 Alexis DA et al. *JAMA Network Open*.

**Belief System**		
Christian	1546 (44)	7811 (57)
Non-Christian	1594 (45)	4106 (30)
**Length of Time at School**		
< 1 year	550 (16)	1871 (14)
1-5 years	1313 (37)	4781 (35)
5-10 years	540 (15)	2620 (19)
≥ 10 years	1085 (31)	4180 (31)
**Position**		
Executive Leadership	131 (4)	431 (3)
Faculty	725 (21)	1475 (11)
Staff	502 (14)	4899 (36)
Student	765 (22)	1177 (9)
Resident/fellow/housestaff	255 (7)	178 (1)
Postdoctoral	199 (6)	192 (1)
**Generational Age Group**		
Traditional (1922-1944)	74 (2)	312 (2)
Baby Boomers (1945-1964)	908 (26)	5458 (40)
Generation X (1965-1980)	1018 (29)	4290 (31)
Millennials (1981-2000)	1447 (41)	3219 (24)
[a]Abbreviation: LGBTQ, Lesbian/Gay/Bisexual/Transgender/Queer		

© 2020 Alexis DA et al. *JAMA Network Open*.
